# Elucidating DNA Damage-Dependent Immune System Activation

**DOI:** 10.3390/ijms26125849

**Published:** 2025-06-18

**Authors:** Elisavet Deligianni, Christina Papanikolaou, Evangelos Terpos, Vassilis L. Souliotis

**Affiliations:** 1 Institute of Chemical Biology, National Hellenic Research Foundation, 116 35 Athens, Greece; edelig@eie.gr (E.D.); chrpapa@eie.gr (C.P.); 2 Department of Clinical Therapeutics, School of Medicine, National and Kapodistrian University of Athens, 115 28 Athens, Greece; eterpos@med.uoa.gr

**Keywords:** DNA-damage response (DDR), immune system, tumor neoantigen burden (TNB), tumor mutational burden (TMB), stimulator of interferon genes (STING), immunogenic cell death (ICD), major histocompatibility complex type I (MHC-I), programmed cell death ligand-1 (PD-L1), immune checkpoint inhibitor

## Abstract

The DNA-damage response (DDR) network and the immune system are significant mechanisms linked to the normal functioning of living organisms. Extensive observations suggest that agents that damage the DNA can boost immunity in various ways, some of which may be useful for immunotherapeutic applications. Indeed, the immune system can be activated by the DDR network through a number of different mechanisms, such as via (a) an increase in the tumor neoantigen burden, (b) the induction of the stimulator of interferon genes pathway, (c) the triggering of immunogenic cell death, (d) an increase in antigen presentation as a result of the augmented expression of the major histocompatibility complex type I molecule, (e) modification of the cytokine milieu in the tumor microenvironment, and (f) altered expression of the programmed cell death ligand-1. Together, the DDR network may improve the effect of immunostimulatory anticancer agents and provide a basis for devising more efficient treatment strategies, such as combinatorial therapies of DDR targeting drugs and immunomodulators. Here, the molecular mechanisms underlying the immune system’s activation by DDR are summarized, along with some of their possible uses in cancer treatment.

## 1. Introduction

The DNA-damage response (DDR) network and the immune system are essential mechanisms for all living organisms. DNA-damage sensors, mediators, transducers and effectors that detect and eliminate different kinds of DNA damage comprise the DDR network [1]. The DDR is activated following the recognition of DNA damage. Subsequently, a sequence of signaling events is initiated, which include cell-cycle checkpoints, DNA-repair pathways and cell death. Indeed, the DDR network controls the cell’s decision to repair damaged DNA or to initiate cell death, a decision that affects how different diseases develop and how well chemotherapy works [2]. Genotoxic substances have been used for extended periods of time in cancer therapy due to their ability to inhibit the replication of cancer cells and/or to induce cell death. Nonetheless, resistance to chemotherapy can arise because of factors related to the host or the tumor. Various approaches could be used to overcome drug resistance, such as changing the dosage of the drugs, optimizing the order in which therapies are administered and using combination therapies to target different molecular mechanisms or to bypass pathways. The fact that cancer cells have a higher proliferation rate and defects in repairing DNA damage makes them more susceptible to targeted inhibition of the DDR. Therefore, DDR inhibitors, a category of drugs capable of altering the DDR network, have recently become a focus of attention in the field of cancer treatment research. DDR inhibitors that are used in clinical trials target molecular components involved in DDR-related pathways, such as non-homologous end joining (NHEJ), DNA-dependent protein kinase (DNA-PK), base excision repair (BER), polyADP-ribose polymerase (PARP), homologous recombination (HR), ataxia telangiectasia and Rad3-related kinase (ATR) and ataxia telangiectasia mutated kinase (ATM), as well as checkpoint kinase 1 (CHK1) and cell-cycle checkpoint kinase WEE1, which are involved in cell-cycle regulation [3]. As for the immune system, this is comprised of cells, tissues and organs that fight infections and diseases. Interestingly, the activation of this system plays a crucial role in the development and progression of cancer, as well as in the outcome of anticancer therapy. Currently, various types of immunomodulators are utilized for cancer treatment, including immune checkpoint inhibitors (ICIs) [4], T-cell transfer therapy [5], monoclonal antibodies [6] and treatment vaccines [7].

Recent data indicate that the DDR network and the immune system collaborate to support the normal functions of multicellular organisms. Multiple studies have shown that a change in the equilibrium of DNA-damage formation and repair, caused by either exposure to high levels of DNA-damaging agents or impairment of DNA-repair mechanisms, triggers the immune system by activating the type I interferon (IFN) and/or the nuclear factor-κB (NF-κB) pathways [8,9,10]. In addition, disruption of the immune balance and prolonged acute inflammation stemming from various triggers, such as aging, infection, dietary factors, toxins, radiation and autoimmune diseases also stimulate the DDR network by causing DNA damage [11,12,13,14]. Indeed, during inflammatory diseases, such as neurological conditions, autoimmune disorders and certain cancers, epithelial and inflammatory cells produce reactive oxygen species (ROS) and reactive nitrogen species (RNS) that lead to the formation of oxidative and nitrative DNA damage and the inhibition of key DNA-repair proteins [2]. Notably, these forms of DNA damage can induce mutations and are thought to play a role in the initiation and/or promotion of inflammation-induced carcinogenesis.

In the past, conventional chemotherapy was believed to be immunosuppressive, with various chemotherapeutics being employed in the treatment of autoimmune disorders [15]. However, increasing evidence indicates that DNA-damaging agents can enhance the immune response in numerous ways, some of which may be utilized for immunotherapy. Here, we present an overview of the molecular mechanisms involved in the DDR-mediated activation of the immune system and describe their potential applications in cancer therapy.

## 2. The Effects of DNA-Damaging Agents on the Immune System

### 2.1. Increased Tumor Mutational Burden (TMB) and the Synthesis of Neoantigens

Neoantigens are newly created antigens produced by tumor cells due to a variety of tumor-specific changes, including genomic mutations, post-translational modifications of proteins and the deregulated splicing of RNA. In virus-associated cancers, such as those associated with the human papilloma virus and Epstein–Barr virus, these may also arise from virally encoded open reading frames [16,17,18]. Neoantigens are unique to tumors, being absent in healthy tissues [19], and they are essential for the effectiveness of different types of immunotherapy, such as personalized tumor vaccines, ICIs and adoptive T-cell transfer immunotherapy [20,21,22]. Neoantigen presentation and load positively correlate with prognosis in various cancers [23,24,25,26], as well as with the efficacy of ICIs in melanoma [27,28], non-small-cell lung cancer (NSCLC) and colorectal cancer with DNA mismatch repair (MMR) deficiency [29]. Interestingly, neoantigens present appealing targets for personalized cancer immunotherapy, since nearly all these tumor antigens vary among patients [19].

As for the TMB, this term refers to the quantitative assessment of mutations that arise in tumor cells [30]. ΤΜΒ is a genetic characteristic that has been correlated with the response to immunotherapy, since a large number of genetic mutations may increase the likelihood of tumor neoantigens and specific T-cell responses [31,32,33,34,35]. Neoantigens produced by a high TMB contribute to the generation of an inflammatory microenvironment, subsequently improving the results following ICI therapy [36]. However, neoantigen levels are not a universal predictive marker for the ICI response. Indeed, while neoantigen levels serve as prognostic indicators for ICI treatment in patients with melanoma and chronic lymphocytic leukemia [37], patients with multiple myeloma show poorer progression-free survival despite a high TMB [38].

Recent data have shown that immunotherapy may be improved by radiation or chemotherapy, possibly due to increased neoantigen presentation [39]. In fact, besides its direct tumoricidal effects, radiation creates an in situ vaccination directly from the irradiated tumor cells [40,41]. For example, neoadjuvant chemoradiotherapy (CRT) changed the hosts’ immune systems and produced novel neoantigen epitopes in locally advanced rectal cancer [42]. In a similar vein, patients with relapsed anal squamous cell carcinoma showed increased TNB levels and better responses to programmed cell death protein 1 (PD-1) inhibitors after CRT [43]. In line with these data, although tumor-infiltrating T-cell levels and programmed cell death ligand 1 (PD-L1) expression are the most widely used biomarkers of the response to PD-1 pathway blockade [44,45,46], according to new findings, the mutational burden and tumor-specific neoantigens may also affect how well immunotherapy works by influencing the tumor’s response to ICIs [38]. Recent data have also shown that deficient DDR-associated pathways in cancer, due to increased exposure to DNA damage and/or reduced DNA-repair capacity, may also increase the effectiveness of immune-based treatments by promoting the production of neoantigens [47,48,49]. Moreover, inhibitors of PARP, ATM and ATR may be able to take advantage of DNA-repair defects in cancer to increase genomic instability [50]. For instance, damaged DNA resulting from PARP inhibitor-associated cytotoxicity may be a source of neoantigens, which would increase the immunogenicity of ovarian tumors [47]. Another study suggested that the combination of PARP and immune checkpoint inhibitors might be a new strategy for treating bladder cancer [51].

Since tumors with a high mutational burden tend to respond more favorably to immune treatments, different approaches have been suggested to convert low-TMB tumors into high-TMB ones. For example, inactivation of MMR raises the mutational burden and causes dynamic profiles, resulting in persistent neoantigen renewal in vitro and in vivo in colorectal, breast and pancreatic mouse cells [52]. Thus, targeting DNA-repair mechanisms can increase the TMB, offering potential therapeutic approaches. Greater neoantigen loads, tumor-infiltrating lymphocyte (TIL) counts and PD-1/PD-L1 expression in immune cells have also been demonstrated in HR-deficient malignancies [25]. According to the evidence, genomic instability causes large neoantigen loads and mutations in tumors, which subsequently induce cells within the tumor microenvironment to increase PD-L1 expression [53].

To conclude, while neoantigens hold significant promise for immunotherapies, other factors, such as DDR pathways, critically affect the mutational burden, neoantigen synthesis and immunotherapy responsiveness. Targeting DDR pathways and utilizing biomarkers like the TMB and the neoantigen load can enhance personalized immunotherapy approaches and improve clinical outcomes across diverse cancer types.

### 2.2. Induction of Immunogenic Cell Death

**Figure 1:**
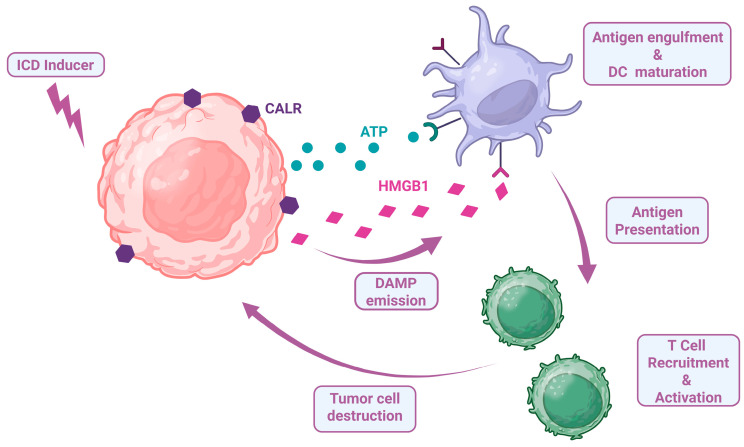
Immunogenic cell death (ICD) and its role in antitumor immunity. Upon exposure to an ICD inducer, tumor cells undergo immunogenic cell death characterized by the release and surface exposure of damage-associated molecular patterns (DAMPs), which include calreticulin (CALR), adenosine triphosphate (ATP) and high-mobility group box 1 (HMGB1). These DAMPs collectively stimulate antigen engulfment and dendritic cell (DC) maturation, enabling effective tumor antigen presentation to T cells. The subsequent T-cell recruitment and activation culminate in a robust antitumor immune response and tumor cell destruction. Figure produced using “BioRender.com (accessed on 23 May 2025)”.

Immunogenic cell death (ICD) refers to a type of cell death characterized by the emission of stress signals, known as danger-associated molecular patterns (DAMPs). Key DAMPs include endoplasmic reticulum chaperones exposed on the plasma membrane of stressed cells, such as calreticulin (CALR), protein disulfide isomerase family A member 3 (PDIA3), heat shock protein 70 kDa (HSP70) and HSP90. Additionally, ICD involves the secretion of adenosine triphosphate (ATP), the production of type I IFNs and pro-inflammatory cytokines like CXC-chemokine ligand 10 (CXCL10), as well as the release of high-mobility group box 1 (HMGB1) and annexin A1 (ANXA1). These signals are detected by antigen-presenting cells (APCs), such as dendritic cells (DCs), which initiate an immune response mediated by cytotoxic T lymphocytes (Figure 1). This process not only leads to the effective elimination of cancer cells but also fosters long-term immune memory that is specific to tumor antigens [54,55,56,57].

ICD can be triggered by a range of cellular stressors such as intracellular pathogens, therapeutic oncolytic viruses, molecules with oncolytic potential, physical therapies (e.g., ionizing radiation and photodynamic therapy) and some conventional chemotherapeutics, including anthracyclines (doxorubicin, epirubicin, idarubicin), topoisomerase II inhibitors (mitoxantrone, teniposide), oxaliplatin, 5-fluorouracil (5-FU), bleomycin, cyclophosphamide and bortezomib [57,58,59]. Interestingly, numerous chemical agents and ionizing radiation that damage DNA can directly destroy tumor cells while simultaneously initiating immunogenic signal transduction. Indeed, Naito and colleagues [60] showed that both oxaliplatin and 5-FU induced CALR exposure in colorectal cancer cells and organoids. Another study discovered that the combination of the ATR inhibitor berzosertib (VE-822) with oxaliplatin showed a synergistic effect in both in vitro and in vivo colorectal cancer models, possibly due to the induction of cytoplasmic DNA and ICD signals, such as CALR, HMGB1 protein and ATP, thus promoting antitumor T-cell responses [61]. Similarly, bleomycin, a naturally derived glycopeptide that inhibits HR repair [62], induces the aforementioned ICD markers [63], thereby triggering the onset of ICD and leading to the induction of IFN-γ and a CD8+ T-cell-mediated immune response against tumor cells.

Moreover, the topoisomerase II inhibitor mitoxantrone induces the release of DAMPs and triggers ICD by activating eukaryotic initiation factor 2α (eIF2α) via upregulation of protein kinase RNA-like ER kinase (PERK)/general control nonderepressible 2 (GCN2) in prostate cancer cells in vitro and induces antitumor immunity in vivo [64]. Moreover, mitoxantrone treatment of colon cancer cells results in the inhibition of G1 cell-cycle progression and an increase in G2/M cell fractions while simultaneously enhancing the dynamic exposure of CALR on the cell surface [65]. Teniposide, another topoisomerase II inhibitor, has also been found to promote the release of HMGB1 and trigger type I IFN signaling in tumor cells [66]. Anthracyclines (doxorubicin and idarubicin) can induce the rapid translocation of CALR, HSP70 and HSP90 to the cell surface, promoting the release of HMGB1 in leukemia, ovarian cancer and prostate cancer cells.

As for bortezomib, this proteasome inhibitor can hinder DNA repair in multiple myeloma cells and trigger ICD [67]. In fact, recent studies reveal that bortezomib can induce the display of CALR on the surface of dying myeloma cells, the uptake of tumor cells by DCs, and the stimulation of a myeloma-specific immune response [68]. Furthermore, Hui and colleagues [69] demonstrated that colorectal cancer cells with a deficiency in B-Myb (a transcription factor implicated in DNA replication and the regulation of the cell cycle) exhibit a higher sensitivity to bortezomib, which significantly amplifies DNA damage and induces cell-cycle arrest. Importantly, bortezomib treatment in B-Myb-deficient cells promotes ICD by increasing the expression of DAMPs such as HMGB1 and HSP90, thus triggering immune activation.

Recent data have shown that identifying ICD- and DDR-related molecular patterns associated with the immune status can be useful for cancer prognosis for intermediate or advanced hepatocellular carcinoma (HCC) [70]. That is, samples derived from HCC patients can be classified into three distinct molecular subtypes with different mutation patterns, various levels of immune cell infiltration and significant differences in their response to immunotherapy. Interestingly, a model based on 11 ICD- and DDR-related genes (ADA, DDX1, DHX58, EIF2AK4, FANCL, FFAR3, MGMT, POLR3G, PI3KR1, SLAMF6, TPT1) provides an important prognostic tool, which can also serve in decision-making for HCC treatment.

Taken together, cytotoxic agents with immunomodulatory potential represent a significant advancement in cancer therapy, as many chemotherapeutic drugs are capable of simultaneously inducing ICD and disrupting DDR networks. This dual action not only enhances direct tumor cell eradication but also activates durable antitumor immune responses through the release of DAMPs. These molecules serve as powerful adjuvants, promoting dendritic cell and cytotoxic T-cell activation, and may also function as diagnostic biomarkers for assessing treatment efficacy. By deepening our understanding of the interplay between ICD and DDR, we can improve the development of immunostimulatory anticancer agents and design more effective treatment strategies.

### 2.3. Activation of the Stimulator of Interferon Genes (STING) Pathway

Under normal cellular homeostatic settings, the genome is found in the nucleus, and dsDNA is absent from the cytoplasm. Therefore, the presence of dsDNA in the cytosol could be a sign of pathogenic dangers or weakened cellular conditions that endanger the host homeostasis. In this situation, maintaining normal host function depends critically on molecular machinery that can identify and communicate possible homeostasis breaches. DNA viruses, retroviruses, intracellular prokaryotes, mitotic defects, DNA rupture debris from the nuclei, mitochondria, as well as various sources of DNA damage, including radiation, oxidative stress, low chromosome instability, hyperactivation of oncogene signaling and chemotherapy, are the primary sources of cytoplasmic DNA (Table 1) [71]. These DNA molecules are either detected by DNA sensors or degraded by TREX1 (three prime repair exonuclease 1) and DNase III (deoxyribonuclease III) in the cytosol. Interestingly, there are multiple ways that radiation and chemotherapy can induce cytosolic DNA, including via (a) direct DNA damage that results in strand breaks and other aberrations [71]; if repair is unsuccessful, fragments with DNA damage can be removed from the nucleus and enter the cytoplasm; (b) nuclear envelope rupture, which allows DNA fragments to escape from the nucleus and leak into the cytoplasm [72]; (c) senescence and the extrusion of cytoplasmic chromatin fragments [73]; (d) phagocytosis, which releases DNA fragments from the phagosomes into the cytoplasm [74]; and (e) mitotic DNA replication stress and chromosome missegregation [75].

**Figure 2:**
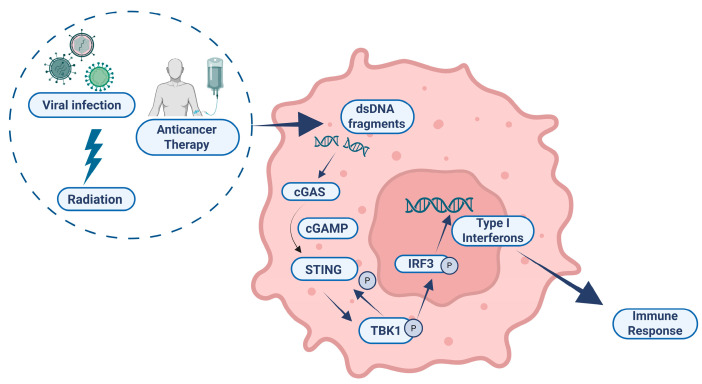
The role of the cGAS-STING pathway in health and cancer therapy. Various stressors such as viral infection, anticancer therapies or radiation can lead to the accumulation of double-stranded DNA (dsDNA) fragments in the cytosol. These fragments are sensed by cyclic GMP–AMP synthase (cGAS), which catalyzes the production of cyclic GMP–AMP (cGAMP). cGAMP binds to and activates the adaptor protein STING (stimulator of interferon genes), triggering its phosphorylation. Activated STING recruits and activates TANK-binding kinase 1 (TBK1), which subsequently phosphorylates the transcription factor IRF3 (interferon regulatory factor 3). Phosphorylated IRF3 translocates into the nucleus, where it induces the expression of type I interferons. These interferons play a critical role in promoting an immune response, contributing to antiviral defense and antitumor immunity. Figure produced using “BioRender.com (accessed on 23 May 2025)”.

The interaction between cytosolic double-stranded DNA (dsDNA) and cyclic guanosine monophosphate–adenosine monophosphate synthase (cGAS) leads to the synthesis of 2′,3′-cyclic GMP–AMP (cGAMP), the production of which is a crucial initial step that triggers antiviral responses in various organisms [85]. Indeed, cGAS can be triggered by exogenous and endogenous nucleic acids that are abnormally localized in the cytosol. The generation of cGAMP triggers the stimulation of STING, which subsequently activates TANK-binding kinase 1 (TBK1), IkB kinase (IKK) and NF-κB inducing kinase (NIK) [85,86]. Next, the activation and movement of the IFN regulatory factor 3 (IRF3) and NF-κB cause the production of type I IFN, interferon-stimulated genes (ISGs) and inflammatory cytokines—further linking the DDR network with the immune system (Figure 2) [87,88].

Changes in the network that respond to DNA damage, whether due to a deficient DNA-repair capacity or exposure to genotoxic substances, could play a role in the STING-induced antitumor immune response. In fact, DDR-deficient (DDRd) tumors exhibited higher levels of IFN-related gene expression and increased amounts of CD4+ and CD8+ T cells in both the tumor and stroma when compared with non-DDRd tumors [89]. Also, elevated levels of IRF3 and TBK1 phosphorylation were detected in breast cancer gene 1/2 (BRCA1/2)-deficient cell lysates compared with BRCA1/2-corrected isogenic lines, with conditioned media from BRCA1/2-mutant or BRCA1/2-depleted cells resulting in the enhanced migration of peripheral lymphocytes. Another study showed that cells from Ataxia–Telangiectasia (AT) patients and *Atm^−/−^* mice exhibited a comparable increase in IFN signaling through STING activation [90].

In addition to being activated under conditions of DNA-repair deficiency, the STING pathway is triggered after chemotherapy treatment, leading to the accumulation of cytoplasmic dsDNA. Indeed, DNA-damaging therapies including cytotoxic chemotherapy (cisplatin, etoposide, mafosfamide, camptothecin, mitomycin C, adriamycin), radiotherapy (RT), ATR and/or PARP inhibitors enhance the levels of DNA-damage-induced cytosolic dsDNA and trigger the cGAS-STING-IFN response, with S-phase DNA damage being a powerful activation factor [89,91,92,93,94]. Activation of the cGAS-STING inflammatory response can also trigger the formation of micronuclei, with the subsequent leaking of DNA from these small nuclei-like structures being able to induce the innate immune response [95,96,97,98].

These findings suggest a broad consensus regarding the significant potential of the cGAS-STING pathway in antitumor therapies. Nevertheless, other research findings indicate that STING may also play a role in tumorigenesis, progression and metastasis. Although chemotherapeutic agents can cause nuclear DNA to leak into the cytoplasm of tumor cells and activate STING-dependent cytokine production, the phagocytic clearance of dead cells subsequently increases the levels of peripheral inflammatory factors in a STING-dependent manner [99]. Indeed, nuclear cGAS can disturb the formation of the PARP–Timeless complex, impede HR, diminish genomic stability, and promote tumor cell development. Other studies have shown that activation of the STING pathway may also facilitate cancer metastasis [100]. In fact, in mesenchymal stromal cells (MSCs), a group of cells that play a crucial role in tumor metastasis, the expression of ISGs, such as the chemokine CCL5, is upregulated alongside the induction of cGAS-STING signaling. Blocking the cGAS-STING pathway in MSCs inhibits these prometastatic effects.

Other studies have also demonstrated that STING might facilitate tumor immune escape. For example, Li and colleagues [101] demonstrated that STING induces regulatory B cells, which suppress the antitumor capacity of natural killer (NK) cells, representing a new mechanism of immune escape. In that study, they used STING agonists to promote the expansion of interleukin (IL)-35-secreting B (IL-35+ B) cells for the production of IL-35 and IL-10 and found that the intratumoral levels of these anti-inflammatory cytokines had a negative relationship with NK cell infiltration and a positive relationship with tumor weight. STING could also induce immunosuppression. For instance, although STING gene expression is upregulated during the progression of tongue squamous cell carcinoma, this activation does not impact cell viability and programmed cell death; rather, it enhances the production of multiple immunosuppressive cytokines, like IL-10, CCL22 and indoleamine 2,3-dioxygenase (IDO1), thus leading to the infiltration of regulatory T cells (Tregs) [102].

Despite these bidirectional immunomodulation results, the direct activation of STING represents an appealing therapeutic approach, with a number of cyclic dinucleotide mimetics that activate STING demonstrating encouraging results in preclinical investigations [103,104]. Because the STING pathway induces type I IFN signaling, tumors lacking baseline type I IFN signaling may be perfect candidates for STING agonist treatment. Indeed, STING agonists have been demonstrated to trigger IFN signaling and extend survival in two acute myeloid leukemia (AML) mouse models where the host type I IFN response was absent [105].

In conclusion, STING activation, triggered by cytosolic DNA sensing, has a dual function in health and cancer therapy. Indeed, STING activation is a crucial part of the innate immune system, which is triggered by the presence of DNA in the cytoplasm, and it plays a central role in health by fighting viral infections and cancer as well as in regulating inflammation and autophagy. In addition, STING activation is a promising strategy in the therapy of cancer due to its role in augmenting antitumor immunity and tumor cell killing by activating the production of type I interferons and pro-inflammatory cytokines.

### 2.4. The Increased Expression of MHC Class I Proteins and the Subsequent Antigen Presentation

The major histocompatibility complex (MHC) class I antigen-presentation pathway enables CD8+ T cells to identify cells producing foreign proteins, such as those from viruses or cancer mutations [106]. During antigen presentation, the ubiquitin-proteasome pathway breaks down proteins into peptides, some of which are transported into the endoplasmic reticulum (ER). Inside the ER, the peptides are further processed and loaded onto newly formed MHC class I molecules with the help of chaperones and peptide editors. The peptide–MHC complex is subsequently delivered to the cell surface, where it fuses with the cell membrane, engendering an immune response. In cells with defects in peptide generation, transport or MHC-I loading, most of their MHC-I molecules are retained in the ER and ultimately degraded, resulting in a reduction in the number of MHC-I molecules on the cell surface. This is a widely employed strategy that allows many cancers to avoid immune recognition [107,108]. Human MHC class I molecules are also called human leukocyte antigens (HLAs).

In many types of cancer, a significant percentage of tumors show partial or complete loss of MHC class I expression, which is usually associated with a poor clinical outcome. Reduced expression of MHC-I is linked to primary resistance to checkpoint inhibition, such as through anti-CTLA-4 and anti-PD-1 therapy. Thus, tumor MHC class I expression status, either alone or in combination with the PD-L1 expression status, may serve as an important factor to consider when selecting patients for immunotherapy [109,110,111,112]. Additionally, β2 microglobulin (B2M), a critical subunit of the HLA class I complex, plays an essential role in surface HLA-I presentation. Indeed, homozygous loss or downregulation of B2M can cause defects in HLA class I antigen processing and presentation, resulting in ICI-resistant tumors [113]. A replication-deficient adenoviral vector encoding the human B2M gene has been developed to boost tumor HLA class I expression, making it a promising nominee for cancer gene therapy [114].

Numerous DNA-damaging drugs affect MHC-I molecule expression through various mechanisms [115]. In fact, different DNA-damaging agents can upregulate HLA-I presentation, irrespective of the type of DNA lesion or the cell type. Gemcitabine, a nucleoside analog that mediates its anticancer activity by triggering apoptosis of cancer cells undergoing DNA synthesis, not only augments HLA-I mRNA transcripts, total protein, surface expression and surface stability in pancreatic cancer cells, but it also improves the quality of the peptide ligands presented by HLA-I [116]. Moreover, cisplatin, etoposide, paclitaxel and vinblastine also induce MHC-I expression by stimulating IFN-β secretion [117]. Topoisomerase inhibition enhances the expression of MHC-I on cancer cells, interfering with NF-κB and DDR pathways by activating type I IFN signaling [117,118]. NF-κB and the lysine acetyltransferases p300 and CREB-binding protein (CBP) are crucial for controlling the MHC-I antigen presentation machinery in human cancers. The chemotherapy drugs oxaliplatin and mitoxantrone activate this pathway even in the absence of IFN-γ signaling, increasing MHC-I expression and antigen presentation. Ablation of NF-κB or p300/CBP disrupts this synergy, preventing chemotherapy-induced MHC-I expression and tumor rejection [119].

RT has cytotoxic properties, inducing irreparable DNA strand breaks in cancer cells. Interestingly, it can also stimulate immune responses, as it changes the expression pattern of several immunomodulatory surface molecules, including MHC-I [120]. It has been proved that RT upregulates nucleotide-binding and oligomerization domain containing 5 (NLRC5), leading to increased MHC-I expression on tumor cells independently of IFN-I or STING signaling [121]. Otherwise, RT causes the release of irradiated tumor-cell-derived microparticles, which induce double-strand breaks (DSBs) and activate the ATM/ATR/CHK1 and the downstream JAK-STAT signaling pathways, leading to the upregulation of MHC-I in non-irradiated tumor cells [122]. Radiation can also amplify MHC class I expression caused by ATM inhibition. This MHC-I upregulation is dependent on the activation of the NF-κB/IRF1/NLRC5 pathway but occurs independently of STING. Therapeutic approaches combining ATM inhibitors with RT and immunotherapy, along with utilizing ATM mutations as potential markers of treatment sensitivity, could result in improved clinical outcomes [123].

Several findings underline the prognostic value of the HLA-I status and its importance in predicting responses following immunotherapy. More specifically, HLA-I loss of heterozygosity (LOH) can serve as a biomarker for predicting the effectiveness of ICIs. Indeed, HLA-I LOH is accompanied by the failure of DNA DSB repair, an increased mutation and neoantigen load, as well as greater subclonal diversity, which can result in a weaker immune response despite higher neoantigen production [115].

To sum up, MHC class I-restricted antigen presentation constitutes a critical mechanism of tumor immune surveillance. Tumors often downregulate MHC-I or disrupt the antigen-processing machinery to escape immune detection. However, emerging evidence shows that DNA-damaging agents and radiotherapy can restore or enhance MHC-I expression. These findings open promising therapeutic avenues, suggesting that combining DNA-damaging treatments with immunotherapy may sensitize resistant tumors and improve patient outcomes. Monitoring associated biomarkers could further refine patient selection for personalized cancer therapies.

### 2.5. Upregulated Expression of PD-L1

**Figure 3:**
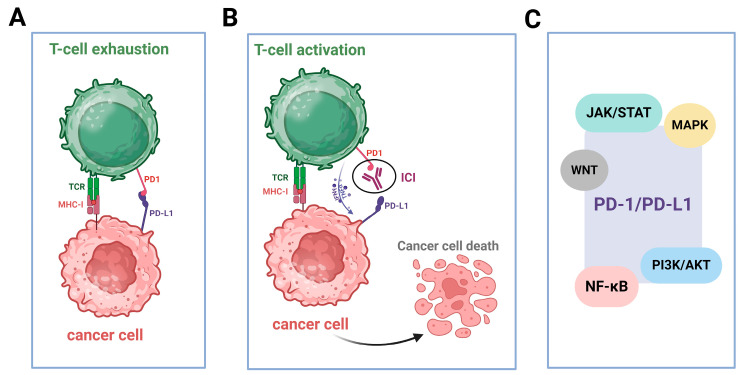
The PD-1/PD-L1 signaling pathway in the onset, progression and management of cancer. (**A**) The interaction of PD-1 and PD-L1 facilitates tumor survival. Specifically, the interaction between PD-1 and its ligand, PD-L1, leads to T-cell and malignant-cell engagement, suppresses downstream T-cell receptor (TCR) signaling and effectively inhibits T-cell activation, which impedes antitumor immune responses. (**B**) Activation of T cells and cancer cell death. The administration of ICIs, such as anti-PD-1 antibodies, can restore the function of exhausted T cells, thereby enhancing their cytotoxic activity and facilitating the elimination of tumor cells. (**C**) Regulation of PD-1/PD-L1 expression by various pathways. Multiple intracellular signaling cascades, including the JAK/STAT, MAPK, WNT, PI3K/AKT and NF-κΒ pathways, are implicated in regulating PD-L1 expression and contributing to tumor immune-evasion mechanisms. Figure produced using “BioRender.com (accessed on 24 May 2025)”.

Immune checkpoints are critical immunosuppressive molecules that regulate immunity, maintain host homeostasis and prevent undesired immune responses under physiological conditions [124]. Among them, the PD-1/PD-L1 axis plays a pivotal role in tumor immune evasion and is a key target in cancer immunotherapy (Figure 3A,B) [125,126]. PD-1, a transmembrane protein of the CD28/CTLA-4 family, is expressed on activated T cells, B cells and monocytes. It exerts its immunosuppressive effects by binding to its ligands, PD-L1 and PD-L2, which are expressed on APCs, tumor cells and other immune and non-hematopoietic cells [16,127,128,129,130]. This interaction delivers inhibitory signals that suppress T-cell activation, reduce effector functions, and promote immune tolerance, ultimately maintaining the immune balance and preventing autoimmunity [131]. PD-1/PD-L1 signaling is essential for peripheral tolerance and immune evasion and inhibits both innate and adaptive responses [132]. PD-L1 is sparingly expressed under normal conditions but is upregulated in tumors, and it is influenced by the IFN-γ secreted by TILs [133,134,135,136]. This creates a feedback loop that impairs antitumor immunity [137]. PD-L1 expression correlates with the prognosis and the therapeutic response [138], yet its predictive value varies across cancers [139]. Pathways like PI3K/AKT, MAPK, JAK/STAT, WNT and NF-κB regulate PD-L1 expression, promoting immune escape (Figure 3C) [140,141,142,143].

It is generally accepted that beyond their cytotoxic effects, RT and chemotherapy modulate the tumor immune microenvironment, including via the induction of PD-L1 expression. In particular, RT is a known inducer of PD-L1 expression, accomplishing this through several mechanisms, including IFN-γ signaling and the epidermal growth factor receptor (EGFR) pathway [144]. When combined with ICIs, RT has been shown to reduce the number of immunosuppressive cells and to enhance cytotoxic T-cell infiltration [145]. It also triggers abscopal responses, which involve the regression of tumors located far from the irradiated area, especially when combined with checkpoint blockades [146]. Preclinical data show that the combination of PD-1/PD-L1 inhibitors with RT increases cytotoxic T lymphocyte (CTL) activity and reduces the frequency of myeloid-derived suppressor cells (MDSCs) [145].

Similarly, chemotherapy with platinum-based drugs (cisplatin, carboplatin, oxaliplatin), antimetabolites (5-FU, decitabine) and alkylating agents (temozolomide, mitomycin C) has off-target immunomodulatory effects. These include enhancing the tumor-antigen availability through cancer cell death and mitigating immunosuppressive signals, thereby promoting tumor-specific T-cell priming [147]. In addition, chemotherapeutic drugs can induce immunogenic cell death, promote tumor-antigen release, and modulate PD-L1 expression through pathways such as ERK1/2, STAT1/3 and DDR-related pathways [53,148,149]. Moreover, the resulting DNA damage generates cytosolic DNA and micronuclei that further activate immune pathways, thus enhancing immunogenicity [50]. Notably, combining chemotherapy with ICIs has shown promise in low-immunogenic tumors, as reported in the KEYNOTE-021 trial, where pembrolizumab (a PD-1 inhibitor) with carboplatin and pemetrexed (an inhibitor of DNA synthesis enzymes) improved NSCLC outcomes [150].

In addition, inhibitors of DDR-related components, such as PARP, ATR, ATM and CHK1/2, enhance immunogenicity by modulating PD-L1 expression [151,152]. This immune activation is further amplified when DDR inhibitors are combined with DNA-damaging approaches like RT or chemotherapy. Such combinations trigger innate and adaptive immune responses and support T-cell recruitment and activation within the tumor microenvironment [87,153]. For example, PARP inhibitors disrupt the repair of single-strand breaks and increase DSBs, leading to a buildup of mutations and neoantigens [154]. These inhibitors upregulate the expression of PD-L1 through cGAS-STING-mediated signaling, thus contributing to immune evasion and sensitizing tumors to PD-1/PD-L1 blockade [155,156]. Moreover, ATR inhibition disrupts cell-cycle checkpoints and stabilizes PD-L1 protein levels [157]. Other studies have shown that inhibition of ATR or its downstream effector, CHK1, can reduce PD-L1 levels by promoting proteasomal degradation, thereby enhancing immune recognition and T-cell-mediated killing [53,156].

Accumulating evidence indicates that tumors with an MMR deficiency (dMMR) commonly overexpress PD-L1 [29,158]. Moreover, HR-deficiency tumors, particularly those with mutations in BRCA1/2, ATM or CHK2, often exhibit high levels of PD-L1 expression, which might correlate with increased efficacy and potential for improved survival when treated with immune checkpoint inhibitors [152,159,160,161,162]. Also, BER deficiency, along with oxidative and replication stress, can upregulate PD-L1 via ATR-CHK1 signaling [149]. In line with these data, DDR inhibitors demonstrate strong synergy with ICIs in tumors characterized by BRCA1/2 loss, MMR deficiency, or other alterations in DDR genes, enhancing T-cell responses and modulating PD-L1 expression [151,152]. Double or triple combinations of ICIs, DDR inhibitors and DNA-damaging drugs are currently under investigation. Indeed, Vendetti and colleagues [163] have shown that AZD6738, an ATR inhibitor, blocks radiation-induced PD-L1 and reduces Treg infiltration, indicating potential strategies for using timing and sequencing to optimize immunotherapy outcomes. Epigenetics, transcription factors and oncogenic pathways (PI3K/AKT, WNT, MAPK) further influence PD-L1 and may support precision therapy [140].

ICIs represent a significant breakthrough in cancer therapy, but their effectiveness as monotherapy differs, and they are not effective for every patient or type of cancer. Considering the relationship between the DDR and the immune system, new clinical trials in solid tumors that integrate DNA damage and/or repair therapies with immunotherapy have shown encouraging results [164]. For instance, combined treatment of the PARP inhibitor olaparib with durvalumab in the OPHELIA phase II trial [165] as well as the use of niraparib and dostarlimab in patients with locally advanced HNSCC treated with radiation [166] demonstrated promising outcomes. Other PARP-inhibitor combinations with immunotherapy have demonstrated encouraging outcomes in other clinical trials [164,167], including olaparib/pembrolizumab in homologous-recombination-deficient (HRD)-positive ovarian cancer and niraparib/dostarlimab in breast cancer [168]. Recurrent/metastatic HNSCC [169] and several other solid tumors, such as triple-negative breast cancer in both the metastatic [170] and neoadjuvant setting [171], metastatic lung cancer [172,173], metastatic esophageal cancer [174] and metastatic bladder cancer [175], can also be treated with combination chemotherapy–immunotherapy regimens.

In conclusion, the intersection of immune checkpoints, the DDR, and traditional therapies is reshaping the immuno-oncology field. PD-1/PD-L1 axis regulation extends beyond T-cell modulation to include DNA repair and tumor evolution. Chemotherapy and radiotherapy, beyond their cytotoxic modalities, function as immune modulators that enhance tumor immunogenicity and help in overcoming resistance to ICIs. Moreover, DDR alterations and the use of DDR inhibitors could create a link between DNA-repair deficiencies and immunotherapy efficacy. Future efforts should focus on optimizing therapeutic combinations and biomarker-guided personalized treatments.

### 2.6. The Induction of a Pro-Inflammatory Milieu in the Tumor Microenvironment (TME)

The tumor microenvironment (TME) is a multifaceted niche that supports tumor development [176]. It comprises stromal cells, including cancer-associated fibroblasts (CAFs), MSCs, endothelial cells (ECs), and pericytes, along with a variety of immune cells such as T and B lymphocytes, NK cells, DCs, tumor-associated macrophages (TAMs), tumor-associated neutrophils (TANs) and MDSCs. It also includes structural and biochemical components such as the extracellular matrix, cytokines, chemokines and other regulatory molecules. Cytokines are a signaling-protein group consisting of interleukins, interferons, tumor necrosis factors, chemokines and growth factors that can either facilitate or suppress tumor progression and regulate the TME. Certain cytokines, including IFN-α, IFN-γ, interleukin-2 (IL-2), IL-12, IL-15 and granulocyte–macrophage colony-stimulating factor (GM-CSF) have anticancer effects, either directly by impeding cell division and triggering apoptosis or indirectly by stimulating an immune response. Conversely, some cytokines, such as epidermal growth factor (EGF), vascular endothelial growth factor (VEGF), transforming growth factor-beta (TGF-β), tumor necrosis factor-alpha (TNF-α), IL-1β, IL-6, colony stimulating factor 1 (CSF-1), C-C motif ligand 2 (CCL2), CCL5 and C-X-C motif chemokine ligand 8 (CXCL8) foster malignant-cell expansion, inducing angiogenesis, enhancing immune evasion, promoting epithelial-to-mesenchymal transition (EMT) and enabling metastasis [177,178,179]. TME components are tightly linked to DNA-repair mechanisms, collectively shaping tumor behavior and impacting cancer progression, immune evasion and treatment resistance.

Radiation-induced DDR can stimulate the secretion of cytokines and chemokines, initiating inflammatory processes and TME alteration. These changes can suppress immune activity, facilitating the invasion and spread of several cancers, such as esophageal squamous cell carcinoma (ESCC) [180]. Indeed, previous studies have found that CXCL1 expression was markedly elevated in CAFs, which contributes to tumor radioresistance by enhancing DNA-damage repair and the MEK/ERK signaling pathway. These results suggest that CAF-secreted CXCL1 may serve as an independent prognostic factor for ESCC patients treated with CRT [181]. On the other hand, Guo and colleagues [182] demonstrated that carbon ion induced significant DNA damage, evidenced by increased γH2AX, 53BP1 and BRCA1 foci. These DNA lesions activated the cGAS-STING pathway, which mediated the secretion of pro-inflammatory cytokines, including IL-12 and IFN-γ, and enhanced immune infiltration by DCs and NKs as well as by CD4+ and CD8+ T cells. Another study explored the effects of inhibiting ATM or ATR in conjunction with RT on head and neck squamous cell carcinoma (HNSCC) [183]. The findings indicate that ATR inhibitors plus RT were more potent in cancer cell elimination than RT alone. Additionally, ATR inhibitors fostered an immunostimulatory response by upregulating inducible costimulator ligand (ICOS-L) and CD137-L surface molecules and amplifying the secretion of pro-inflammatory cytokines such as IL-6 and IL-8, whereas ATM inhibitors appeared to dampen immune responses. Interestingly, Chen and colleagues [184] uncovered a cancer-promoting role for the ATM kinase, proving that ATM activation by oxidative stress, rather than DNA damage, drives the expression of IL-8, a pro-inflammatory cytokine that enhances cancer cell migration and invasion. ATR inhibitors, in combination with radiation, were also associated with a higher presence of CD3+ T cells, NK cells, DCs and myeloid cells. Additionally, it triggers a type I/II IFN response and increases CCL2, CCL5 and CXCL10 cytokine levels, contributing to further immune cell recruitment. However, this therapy leads to an increased presence of immunosuppressive cells, such as TAMs and CD11b+ Gr1+ myeloid cells [185].

Moreover, chemotherapy against acute lymphoblastic leukemia triggers the NF-κB transcription factor p65, which directly modulates cytokine expression [186]. Indeed, this cascade resulted in the release of several cytokines, like growth differentiation factor 15 (GDF15), CCL3 and CCL4, which facilitated bone-marrow niche reconstruction, offering a protective environment for the remaining leukemia cells post-chemotherapy. Previous research showed that pericytes facilitate the resistance to temozolomide by releasing CCL5, which binds to the chemokine receptor CCR5 on tumor cells, triggering the DNA-PKcs/AKT pathway to enhance DNA repair and diminish cell death [187].

It is widely accepted that senescence-induced microenvironmental alterations contribute to cancer progression by sustaining DNA damage and inflammation [188]. In fact, senescent cells promote DDR activation in neighboring proliferating cells, as evidenced by the increased phosphorylation of γH2AX, ATM, Chk2 and p53, leading to cell cycle arrest. This occurs through the Senescence-Associated Secretory Phenotype (SASP), which results in the hypersecretion of critical inflammatory cytokines, such as IL-1β, IL-6, IL-8 and TGFβ. These cytokines drive ROS production, further fueling the DDR in nearby cells. Furthermore, while senescence prevents the proliferation of damaged cells, it paradoxically promotes inflammation through the secretion of cytokines like IL-6 and IL-8. This secretion is driven by sustained DDR activity, particularly through ATM, NBS1 and CHK2, independently of p53 and retinoblastoma protein. Interestingly, DDR-driven cytokine secretion enhances cancer cell invasion, contributing to tumor progression and age-related pathologies [189].

Together, the intricate interplay between the DDR and the TME is decisive for cancer progression, immune evasion and therapeutic resistance. While DDR activation can trigger antitumor immune responses, it can also foster an immunosuppressive environment, depending on the context and nature of the signals involved. Persistent inflammation, cytokine signaling and oxidative stress create a feedback loop that sustains genomic instability, fueling tumor evolution and enhancing the cell’s ability to escape immune surveillance. Understanding these complex interactions provides valuable insights into potential therapeutic strategies that target not only tumor cells but also the broader microenvironmental context to overcome resistance and to achieve durable responses.

## 3. Conclusions

**Figure 4:**
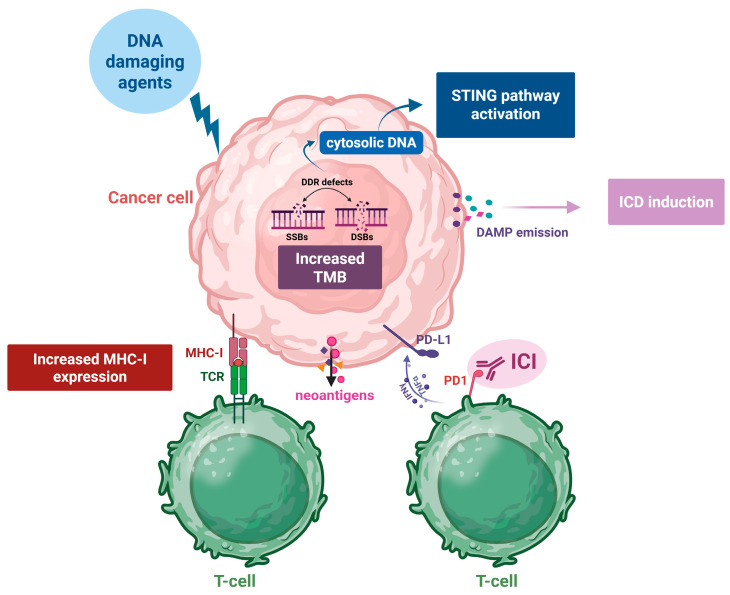
DDR-induced immunogenic modulation. DNA-damaging agents induce a variety of DNA lesions, and if repair is unsuccessful, they may increase the tumor mutational burden (TMB). An elevated TMB promotes the generation of neoantigens, which are presented on MHC class I molecules to T-cell receptors (TCR), enhancing T-cell recognition. DNA damage also results in cytosolic DNA accumulation, triggering STING pathway activation and the release of damage-associated molecular patterns (DAMPs), thereby promoting immunogenic cell death (ICD). Concurrently, cancer cells may upregulate PD-L1 expression to evade immune responses through a PD-1/PD-L1 interaction. Immune checkpoint inhibitors (ICIs) block this pathway, restoring T-cell function and reinforcing their antitumor activity. This illustration highlights how DDR-targeting therapies can synergize with ICIs to potentiate cancer immunotherapy. Figure produced using “BioRender.com (accessed on 23 May 2025)”.

A growing body of evidence suggests that there is crosstalk between the DDR network and the immune system. Although this intricate interplay contributes to the well-being of every living organism, it is also implicated in the pathogenesis and progression of several diseases, including cancer, as well as in the response to therapeutic interventions. Recent data have shown that the DDR can activate the immune system through a number of different molecular mechanisms that could be leveraged to improve patient outcomes, serve as diagnostic tools or act as biomarkers to assess treatment efficacy (Figure 4). Therefore, future research should focus on elucidating the molecular details of the DDR and immune system interaction in order to enhance the development of immunostimulatory anticancer agents; optimize efficient treatment options, including combinatorial therapies of DDR targeting drugs and immunomodulators; and define biomarker-driven strategies to guide clinical decision-making in the evolving field of personalized oncology.

## Figures and Tables

**Table 1:** Sources of cytoplasmic DNA.

Sources		Ref.
External factors	virus	[76]
	retrovirus	[77]
	bacteria	[78]
	oxidative stress	[79]
	chemotherapy	[80]
	radiation	[81]
Internal factors	damaged nuclear DNA	[71]
	mitochondrial DNA	[71]
	mitotic defects	[71]
	DNA from micronuclei	[71]
	hyperactivation of oncogene signaling	[82]
	low chromosome instability	[83]
	cell debris	[84]
